# Everyone needs good neighbours – the intricate relationship between the acetylcholine-receptor channel and its membrane environment

**DOI:** 10.1107/S2052252517009058

**Published:** 2017-06-29

**Authors:** Stephen P. Muench

**Affiliations:** a School of Biomedical Sciences, Faculty of Biological Sciences, University of Leeds, Leeds LS2 9JT, UK; b Astbury Centre for Structural and Molecular Biology, University of Leeds, Leeds, LS2 9JT UK

**Keywords:** acetylcholine receptor, cholesterol, lipid microdomain, cryo-EM, helical image reconstruction, membrane proteins

## Abstract

A short commentary is given on the article in this issue by Nigel Unwin [*IUCrJ* (2017), **4**, 393–399] on ‘Segregation of lipids near acetylcholine-receptor channels imaged by cryo-EM’.

The field of electron microscopy (EM) has undergone significant advancement in its capabilities in terms of both the resolution obtainable and the diverse range of systems that can be studied (Subramaniam *et al.*, 2016 ▸). A particular strength of EM has been in determining the structure of membrane proteins, which have proven to be highly challenging owing to the difficulties in overexpressing them and their instability outside of the membrane environment (Rawson *et al.*, 2016 ▸). This is a significant hurdle in structure-based drug design and target validation with ∼60% of drugs targeting membrane proteins, for example G-protein coupled receptors (GPCRs), ion channels and ABC transporters (Overington *et al.*, 2006 ▸).

One of the best characterized membrane proteins is the acetyl choline (ACh) receptor, a ligand-gated ion channel which consists of a pentamer of protein subunits (β, δ, α_δ_, γ, α_γ_) (Fig. 1 ▸). Through a series of landmark time-resolved EM experiments the mechanism of traversing the channel has been elucidated, whereby binding of acetyl­choline to α_γ_ causes a displacement of β, which in turn results in an opening of the pore to allow for the passage of Na^+^ ions (Unwin & Fujiyoshi, 2012 ▸; Miyazawa *et al.*, 2003 ▸). This movement of charge is sufficient to initiate an action potential through depolarizing the postsynaptic membrane. However, what is more poorly understood is the role that the surrounding lipidic environment plays in the action of the ACh receptor. This has important implications as the lipid environment has been shown to be involved in catalytic cycling, transport, regulation and/or stability. For example, the β-barrel membrane protein OmpX, has been shown to have pronounced dynamic variability in different lipid environments and detergents (Frey *et al.*, 2017 ▸), and cardiolipin can promote dimerization of membrane proteins (Gupta *et al.*, 2017 ▸). Therefore, it is becoming more important, when studying membrane proteins, that a near-native environment is maintained where possible. This can be partially achieved through approaches such as nanodiscs, which maintain a bilayer environment, or styrene maleic acid copolymers (SMALPs), which extract membrane proteins with their native lipids, but extraction in the native membrane itself is the gold standard (Gao *et al.*, 2016 ▸ & Lee *et al.*, 2016 ▸).

To this end, the study by Nigel Unwin (Unwin, 2017 ▸) in this issue of **IUCrJ** has advanced our understanding of the ACh receptor by imaging and reconstructing it in the unperturbed natural membrane. Through advances in the EM field, including more sensitive detectors and stable microscopes, the ACh receptor is examined in a more native environment taking account of the local lipid environment. Two-dimensional classification was used to sort the data, with the best class resulting in a final resolution of 8.4 Å. By using a single-particle helical method as opposed to a Fourier–Bessel helical method, the membrane region is better defined. This shows that in several locations, immediately adjacent to the protein surface, there is a weakening or loss of density. These low-density patches form a microdomain (∼26 Å × 11 Å) enclosed by helices M1 and M4 and polar phospho­lipid headgroups. Smaller patches are also present next to the β, α_γ_, γ, α_δ_ subunits and the M1 and M4 helices. This loss of density can be attributed to the presence of cholesterol which has a much smaller headgroup than other lipids and is present in larger amounts in the native membrane (∼35 mol%), compared with smaller phospho­lipids such as phosphatidic acid. The presence of cholesterol around the ACh receptor is not a new observation, indeed it had been previously reported (Barrantes, 2010 ▸). However, what sets this study apart is the ability to structurally characterize the location of these rafts around the ACh receptor for the first time. But what role does cholesterol play in ACh-receptor function? It has been suggested within this study that for the β subunit to achieve an outward tilt, opening up the channel, the δ subunit must resist the force. The cholesterol-rich microdomain would reduce the free volume available for molecular motion and thus impose resistance and rigidity next to the δ subunit. This hypothesis is yet to be proven but opens up the next chapter in understanding ACh-receptor structure and function, with improved EM resolution, time-resolved studies, and the use of biochemical and mass spectrometry lipidomic analysis the role of the native lipidic environment will be better understood, providing new insights into this essential membrane protein.

## Figures and Tables

**Figure 1:**
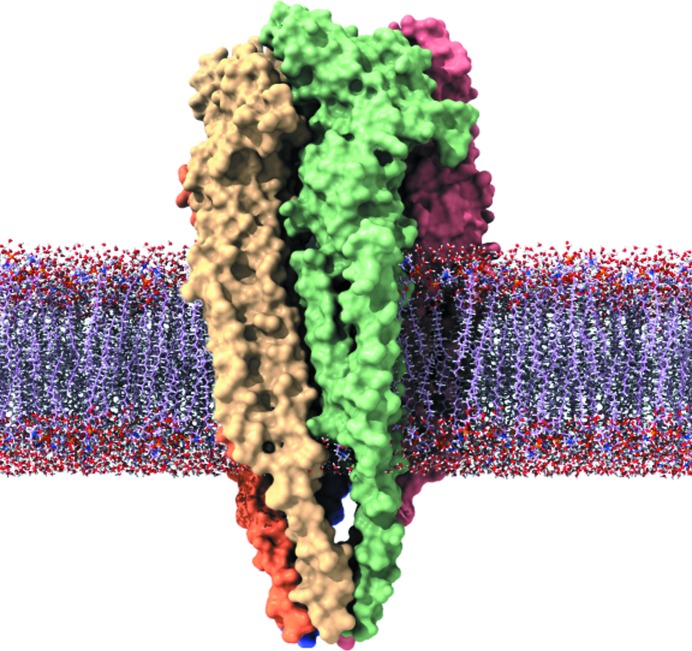
The ACh receptor housed within the membrane environment. The five different subunits are shown with an orange, yellow, green, blue and red surface (note the blue subunit is largely occluded by the other four subunits). The structure was generated from the PDB file (PDB reference: 4aq5) (Unwin & Fujiyoshi, 2012 ▸).
